# Practical Notes on Diagnosis and Treatment

**Published:** 1907-03-16

**Authors:** 


### PRACTICAL NOTES ON DIAGNOSIS AND TREATMENT.

### Vaginitis.

A FIVE-per cent. solution of methyl-blue applied on tampons is said to act directly upon the bacteria in vaginitis, and to cause prompt cessation of the pain and discharge.

### Stricture of the Rectum.

NON-MALIGNANT stricture is much more common in women than in men—in a proportion of at least three to one—and in many instances, perhaps in the majority, the disease is of syphilitic origin.—*Sir William MacCormac.*

### Sub-phrenic Abscess.

IT has been said that the heart's apex is not displaced by a sub-phrenic abscess. But this is not universally correct. The fact is that the apex beat is sometimes considerably displaced, whilst in other instances it is not displaced at all.—*Dr. Gee.*

### Cardiac Disease and Amenorrhœa.

CONTRARY to the general belief, organic cardiac disease is usually associated with *scanty* menstruation, and finally, in many cases, with absolute amenorrhœa, rather than with menorrhagia.—*Dr. Jno. Phillips.*

### Examination of the Prostate.

WITH the finger in the rectum the apex of the prostate gland is found about 1½ in. from the anal orifice. By introducing the finger for another inch the base of the gland when normal can be felt. A well-known surgeon used to advise his students never to lose an opportunity of examining a patient per rectum.

### Quinine in Pregnancy.

As the result of an inquiry conducted some years ago by the Obstetrical Society, it was shown that quinine does not in any way interfere with gestation, and that it neither precipitates nor retards labour; also that, given during the first few days of the puerperium, quinine has a salutary effect on keeping up the tonic contraction of the uterus.

### Inflammation over the Mastoid.

PRIMARY inflammation of the periosteum of the mastoid process may be treated by the application of leeches, or by cold continuously applied. But if there is high fever it is best at once to make an incision right down to the bone dividing the periosteum freely. Begin the incision from below and cut upwards, keeping as close behind the ear as possible so as to avoid any obtrusive scar.—*Dr. Wm. C. Bull.*

### Sub-involution of the Uterus.

As regards medicines, ergot is the sheet anchor, though purgatives and especially magnesium sulphate, are an essential part of the treatment. An acid tonic, for instance, with ergot and magnesium sulphate generally answers particularly well. In advanced cases where there is induration of the uterus a little mercury is desirable. The following is a favourite pill: Ergotin gr. 1-1½, hydrarg. perchlor. gr. ⅛, ext. aloes aquos gr. ½-1, ext. nucis vom., gr. ½, ext. belladon., gr. ⅛. A pill to be taken night and morning.

### Psoriasis of the Scalp.

DR. BULKLEY recommends the following ointment to be applied twice daily: White precipitate, bismuth carbonate, of each ʒss., carbolic acid min. x, vaselin ʒij. Frequent washing should be avoided.

### Bismuth in Gastric Catarrh.

DR. PICK urges that the usual dose of 15 grains or so of bismuth subnitrate is largely useless. In the treatment of chronic gastric catarrh he prescribes a teaspoonful or less of Carlsbad salt dissolved in half a pint of warm water. Half an hour later he orders a heaped-up teaspoonful of bismuth subnitrate, dividing this into two doses, and administering it in wafer paper. The gastric region is then gently massaged, to bring the bismuth into contact with all parts of the mucous surface.

### Sweating Feet.

FOR this complaint Hebra used an ointment made by mixing equal parts of linseed oil and melted diachylon plaster. This, after the feet have been washed and dried, is applied on pieces of lint inserted between the toes, and the whole foot is wrapped in lint covered with the ointment. Soft woollen stockings are then put on, and thin low shoes. After the dressing has been repeated twice daily for a few days a thick cuticle is exfoliated, leaving a healthy skin. Then the use of an astringent dusting-powder is continued for some days or weeks.

